# Clostebol acetate

**DOI:** 10.1107/S1600536811026560

**Published:** 2011-07-09

**Authors:** Elisabetta Maccaroni, Andrea Mele, Renato Del Rosso, Luciana Malpezzi

**Affiliations:** a Department of Chemistry, Materials and Chemical Engineering "G. Natta", Politecnico di Milano, Via Mancinelli 7, I-20131 Milano, Italy

## Abstract

The title compound, C_21_H_29_ClO_3_ [systematic name (8*R*,9*S*,10*R*,13*S*,14*S*,17*S*)-4-chloro-3-oxoandrost-4-en-17β-yl acetate],  is a 4-chloro derivative of testosterone, used as an anabolic androgenic agent or applied topically in ophthalmological and dermatological treatments. The absolute configurations at positions 8, 9, 10, 13, 14 and 17 were established by refinement of the Flack parameter as *R*, *S*, *R*, *S*, *S*, and *S*, respectively. Rings *B* and *C* of the steroid ring system adopt chair conformations, ring *A* has a half-chair conformation, while ring *D* is in a C_13_ envelope conformation. Ring *B* and *C*, and *C* and *D* are *trans* fused. In the crystal, molecules are linked by a weak C—H⋯O interaction.

## Related literature

For the characterization of related structures, see Duax *et al.* (1971 ▶); Böcskei *et al.* (1996 ▶); Verma *et al.* (2006 ▶). For the synthesis by direct (or *via* epoxide) chlorination of the 4 carbon atom of the testosterone mol­ecule, see: Camerino *et al.* (1956 ▶); Julian Laboratories Inc. Illinois (1960 ▶); Società Farmaceutici Italia (1960 ▶). For physiological properties when used topically in dermatological and ophthalmological treatments and by application of an anabolic drug, see: Sweetman (2009 ▶). For standard bond lengths, see: Allen *et al.* (1987 ▶) and for ring puckering parameters, see: Cremer & Pople (1975 ▶).
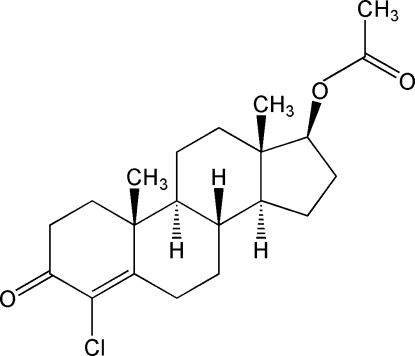


## Experimental

### 

#### Crystal data

C_21_H_29_ClO_3_
*M* *_r_* = 364.89
Orthorhombic, 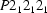

*a* = 7.740 (1) Å
*b* = 12.631 (2) Å
*c* = 19.275 (2) Å
*V* = 1884.4 (4) Å^3^
*Z* = 4
Mo *K*α radiation
μ = 0.22 mm^−1^
*T* = 295 K
0.26 × 0.11 × 0.10 mm

#### Data collection

Bruker SMART APEX CCD area-detector diffractometer
Absorption correction: multi-scan (*SADABS*; Sheldrick, 1996 ▶) *T*_min_ = 0.945, *T*_max_ = 0.978
8471 measured reflections
3325 independent reflections
2586 reflections with *I* > 2σ(*I*)
*R*_int_ = 0.032

#### Refinement

*R*[*F* ^2^ > 2σ(*F* ^2^)] = 0.038
*wR*(*F* ^2^) = 0.079
*S* = 0.97
3325 reflections
242 parameters
H atoms treated by a mixture of independent and constrained refinement
Δρ_max_ = 0.17 e Å^−3^
Δρ_min_ = −0.15 e Å^−3^
Absolute structure: Flack (1983 ▶), 1392 Friedel pairs
Flack parameter: −0.02 (6)

### 

Data collection: *SMART* (Bruker, 2003 ▶); cell refinement: *SAINT* (Bruker, 2003 ▶); data reduction: *SAINT*; program(s) used to solve structure: *SIR97* (Altomare *et al.*, 1999 ▶); program(s) used to refine structure: *SHELXL97* (Sheldrick, 2008 ▶); molecular graphics: *SHELXTL/NT* (Sheldrick, 2008 ▶); software used to prepare material for publication: *SHELXL97*.

## Supplementary Material

Crystal structure: contains datablock(s) global, I. DOI: 10.1107/S1600536811026560/zl2382sup1.cif

Structure factors: contains datablock(s) I. DOI: 10.1107/S1600536811026560/zl2382Isup2.hkl

Additional supplementary materials:  crystallographic information; 3D view; checkCIF report

## Figures and Tables

**Table 1:** Hydrogen-bond geometry (Å, °)

*D*—H⋯*A*	*D*—H	H⋯*A*	*D*⋯*A*	*D*—H⋯*A*
C6—H6*B*⋯O1^i^	0.97	2.62	3.565 (3)	166

Symmetry code: (i) 
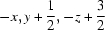
.

**Fig. 1.**
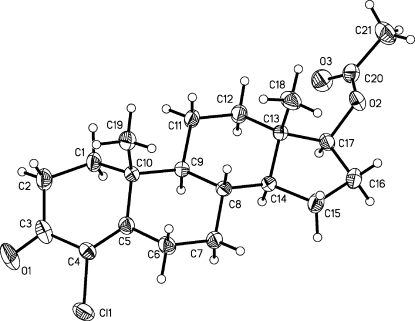
Molecular structure of clostebol acetate with the atomic numbering. Displacement ellipsoids are drawn at the 30% probability level.

**Fig. 2.**
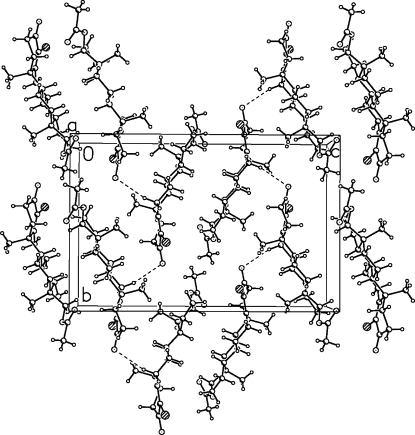
Crystal packing of clostebol acetate viewed along the a axis. Intermolecular C—H···O weak interactions are shown by dashed lines.

**Fig. 3.**
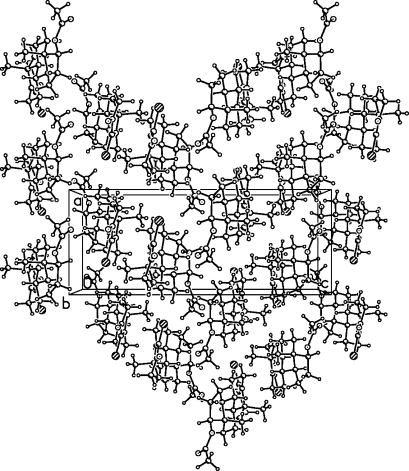
Crystal packing of clostebol acetate viewed along the b axis.

## supplementary crystallographic information

### Comment

Clostebol acetate (Fig. 1), systematic name: (8*R*,9*S*,10*R*,13*S*,14*S*,17*S*)-4-chloro-3-oxoandrost-4-en-17β-yl acetate, is a 4-chloro derivative of testosterone, used as an anabolic androgenic agent or applied topically in ophthalmological and dermatological treatments (Sweetman, 2009). The dermal preparations of this steroid are usually used in the treatment of wounds and ulcers. The absolute configuration has been determined by refinement of the Flack parameter (Flack, 1983) which converged to -0.02 (6). Ring B and C, and C and D are *trans* fused. Ring A (C1—C5, C10) of the steroid ring system adopts a half chair conformation with puckering parameters (Cremer and Pople, 1975) of Q (total puckering amplitude) = 0.466 (3) Å, θ (azimuthal angle) = 56.4 (4)°, φ (phase angle) = 20.0 (4)°. Ring B (C5—C10) and C (C8, C9, C11—C14) are in chair conformations [ring B: Q = 0.543 (2) Å, θ = 2.0 (2)°, φ = 150 (6)°; ring C: Q = 0.575 (2) Å, θ = 3.4 (2)°, φ = 295 (3)°]. The five-membered ring D (C13—C17) is in a C_13_ envelope conformation with puckering amplitude q_2_ =0.455 (2)Å and phase angle φ_2_ = 189.0 (3)°. Bond lengths and valency angles are within the range of expected values for these types of compounds (Allen *et al.*, 1987, Böcskei *et al.*, 1996, Duax *et al.*, 1971, Verma *et al.*, 2006). The acetate group is equatorially attached to the D ring and its orientation may be described by the torsion angle C17—O2—C20—O3 (0.1 (3)°).

In the crystal the molecules are linked by C—H···O weak interactions [C6—H6B···O1 = 166°; C6···O1 = 3.565 (3) Å; H6B···O1= 2.62 Å] to form chains in a herringbone arrangement running parallel to the *b* axis (Fig. 2).

### Experimental

The title compound was obtained by direct or *via* epoxide chlorination on the carbon atom in the 4 position of the testosterone acetate (Camerino *et al.*, 1956, Julian Laboratories, 1960, Società Farmaceutici Italia, 1960). The purification of the crude product was carried out by selective crystallization. Single crystals were obtained from a methanol supersaturated solution at ambient temperature.

### Refinement

H atoms were positioned geometrically and refined in a riding model, except those bonded to the asymmetric carbon atoms, whose positions were freely refined. All H atoms were refined with *U*_iso_(H) values equal to 1.5 *U*_eq_ of the carrier atom for methyl groups and 1.2 *U*_eq_ for all remaining C atoms.

### Crystal data

**Table:**

C_21_H_29_ClO_3_	*D*_x_ = 1.286 Mg m^−^^3^
*M**_r_* = 364.89	Mo *K*α radiation, λ = 0.71069 Å
Orthorhombic, *P*2_1_2_1_2_1_	Cell parameters from 3284 reflections
*a* = 7.740 (1) Å	θ = 2.7–24°
*b* = 12.631 (2) Å	µ = 0.22 mm^−^^1^
*c* = 19.275 (2) Å	*T* = 295 K
*V* = 1884.4 (4) Å^3^	Prism, colourless
*Z* = 4	0.26 × 0.11 × 0.10 mm
*F*(000) = 784	

### Data collection

**Table:**

Bruker SMART APEX CCD area-detector diffractometer	3325 independent reflections
Radiation source: fine-focus sealed tube	2586 reflections with *I* > 2σ(*I*)
graphite	*R*_int_ = 0.032
φ and ω scans	θ_max_ = 25.1°, θ_min_ = 1.9°
Absorption correction: multi-scan (*SADABS*; Sheldrick, 1996)	*h* = −7→9
*T*_min_ = 0.945, *T*_max_ = 0.978	*k* = −13→14
8471 measured reflections	*l* = −23→17

### Refinement

**Table:**

Refinement on *F*^2^	Hydrogen site location: inferred from neighbouring sites
Least-squares matrix: full	H atoms treated by a mixture of independent and constrained refinement
*R*[*F*^2^ > 2σ(*F*^2^)] = 0.038	*w* = 1/[σ^2^(*F*_o_^2^) + (0.0396*P*)^2^] where *P* = (*F*_o_^2^ + 2*F*_c_^2^)/3
*wR*(*F*^2^) = 0.079	(Δ/σ)_max_ = 0.035
*S* = 0.97	Δρ_max_ = 0.17 e Å^−^^3^
3325 reflections	Δρ_min_ = −0.15 e Å^−^^3^
242 parameters	Extinction correction: *SHELXL97* (Sheldrick, 2008), Fc^*^=kFc[1+0.001xFc^2^λ^3^/sin(2θ)]^-1/4^
0 restraints	Extinction coefficient: 0.0024 (7)
Primary atom site location: structure-invariant direct methods	Absolute structure: Flack (1983), 1392 Friedel pairs
Secondary atom site location: difference Fourier map	Flack parameter: −0.02 (6)

### Special details

**Table:**

Geometry. All e.s.d.'s (except the e.s.d. in the dihedral angle between two l.s. planes) are estimated using the full covariance matrix. The cell e.s.d.'s are taken into account individually in the estimation of e.s.d.'s in distances, angles and torsion angles; correlations between e.s.d.'s in cell parameters are only used when they are defined by crystal symmetry. An approximate (isotropic) treatment of cell e.s.d.'s is used for estimating e.s.d.'s involving l.s. planes.
Refinement. Refinement of *F*^2^ against ALL reflections. The weighted *R*-factor *wR* and goodness of fit *S* are based on *F*^2^, conventional *R*-factors *R* are based on *F*, with *F* set to zero for negative *F*^2^. The threshold expression of *F*^2^ > σ(*F*^2^) is used only for calculating *R*-factors(gt) *etc*. and is not relevant to the choice of reflections for refinement. *R*-factors based on *F*^2^ are statistically about twice as large as those based on *F*, and *R*- factors based on ALL data will be even larger.

### Fractional atomic coordinates and isotropic or equivalent isotropic displacement parameters (Å^2^)

**Table:**

	*x*	*y*	*z*	*U*_iso_*/*U*_eq_	
Cl1	−0.18920 (8)	0.39038 (5)	0.85472 (3)	0.0597 (2)	
O1	0.1184 (2)	0.26483 (14)	0.84059 (11)	0.0792 (6)	
O2	0.39800 (19)	1.08493 (11)	0.95249 (8)	0.0469 (4)	
O3	0.6176 (2)	1.00534 (15)	1.00740 (9)	0.0630 (5)	
C1	0.3598 (3)	0.50564 (17)	0.83239 (14)	0.0493 (7)	
H1A	0.4714	0.5283	0.8150	0.059*	
H1B	0.3671	0.5025	0.8826	0.059*	
C2	0.3202 (3)	0.39468 (18)	0.80438 (14)	0.0568 (7)	
H2A	0.3219	0.3961	0.7541	0.068*	
H2B	0.4088	0.3458	0.8198	0.068*	
C3	0.1487 (3)	0.35718 (19)	0.82862 (13)	0.0521 (7)	
C4	0.0152 (3)	0.43994 (17)	0.83408 (11)	0.0392 (6)	
C5	0.0422 (3)	0.54305 (17)	0.82283 (12)	0.0386 (6)	
C6	−0.1033 (3)	0.62146 (17)	0.81864 (13)	0.0486 (6)	
H6A	−0.2112	0.5863	0.8298	0.058*	
H6B	−0.1118	0.6483	0.7716	0.058*	
C7	−0.0757 (3)	0.71354 (17)	0.86839 (14)	0.0471 (6)	
H7A	−0.1674	0.7650	0.8620	0.057*	
H7B	−0.0820	0.6878	0.9157	0.057*	
C8	0.0979 (2)	0.76714 (16)	0.85698 (12)	0.0341 (5)	
H8	0.101 (3)	0.7987 (16)	0.8113 (11)	0.041*	
C9	0.2445 (2)	0.68539 (16)	0.86173 (12)	0.0343 (5)	
H9	0.240 (2)	0.6573 (16)	0.9119 (11)	0.041*	
C10	0.2233 (2)	0.58776 (17)	0.81189 (11)	0.0368 (5)	
C11	0.4216 (3)	0.73900 (17)	0.85446 (14)	0.0517 (7)	
H11A	0.4337	0.7654	0.8075	0.062*	
H11B	0.5112	0.6865	0.8619	0.062*	
C12	0.4487 (3)	0.83096 (17)	0.90544 (14)	0.0490 (7)	
H12A	0.4536	0.8035	0.9524	0.059*	
H12B	0.5581	0.8653	0.8956	0.059*	
C13	0.3036 (3)	0.91174 (16)	0.90011 (10)	0.0342 (5)	
C14	0.1318 (3)	0.85315 (16)	0.91032 (12)	0.0354 (5)	
H14	0.140 (3)	0.8154 (16)	0.9553 (12)	0.043*	
C15	0.0001 (3)	0.94195 (17)	0.92036 (15)	0.0553 (7)	
H15A	−0.0970	0.9178	0.9480	0.066*	
H15B	−0.0425	0.9672	0.8760	0.066*	
C16	0.1004 (3)	1.02920 (19)	0.95832 (15)	0.0545 (7)	
H16A	0.0902	1.0960	0.9339	0.065*	
H16B	0.0569	1.0382	1.0051	0.065*	
C17	0.2886 (3)	0.99196 (17)	0.95951 (12)	0.0395 (5)	
H17	0.325 (3)	0.9551 (17)	1.0049 (11)	0.047*	
C18	0.3114 (4)	0.97189 (19)	0.83157 (12)	0.0572 (7)	
H18A	0.2194	1.0228	0.8299	0.086*	
H18B	0.2992	0.9230	0.7938	0.086*	
H18C	0.4203	1.0077	0.8279	0.086*	
C19	0.2448 (3)	0.6192 (2)	0.73547 (11)	0.0513 (7)	
H19A	0.2299	0.5579	0.7067	0.077*	
H19B	0.3582	0.6480	0.7284	0.077*	
H19C	0.1597	0.6714	0.7235	0.077*	
C20	0.5581 (3)	1.0808 (2)	0.97850 (13)	0.0477 (6)	
C21	0.6504 (4)	1.1833 (2)	0.96711 (16)	0.0741 (9)	
H21A	0.7083	1.2039	1.0091	0.111*	
H21B	0.5685	1.2370	0.9543	0.111*	
H21C	0.7337	1.1750	0.9306	0.111*	

### Atomic displacement parameters (Å^2^)

**Table:**

	*U*^11^	*U*^22^	*U*^33^	*U*^12^	*U*^13^	*U*^23^
Cl1	0.0606 (4)	0.0525 (4)	0.0660 (4)	−0.0228 (3)	0.0059 (3)	0.0004 (3)
O1	0.0913 (14)	0.0317 (10)	0.1147 (17)	−0.0053 (9)	−0.0191 (12)	0.0070 (11)
O2	0.0526 (10)	0.0304 (9)	0.0577 (10)	−0.0054 (8)	−0.0080 (8)	−0.0005 (8)
O3	0.0560 (11)	0.0604 (12)	0.0725 (13)	0.0045 (10)	−0.0090 (9)	0.0027 (10)
C1	0.0381 (13)	0.0344 (13)	0.0755 (18)	0.0063 (10)	0.0005 (11)	−0.0100 (13)
C2	0.0511 (14)	0.0368 (13)	0.0826 (19)	0.0094 (13)	−0.0048 (14)	−0.0104 (14)
C3	0.0690 (18)	0.0332 (15)	0.0541 (16)	−0.0028 (12)	−0.0187 (13)	−0.0016 (12)
C4	0.0418 (13)	0.0367 (14)	0.0392 (14)	−0.0070 (11)	−0.0041 (11)	−0.0010 (11)
C5	0.0404 (13)	0.0350 (13)	0.0403 (14)	−0.0021 (10)	−0.0017 (10)	−0.0045 (11)
C6	0.0339 (13)	0.0388 (13)	0.0732 (17)	−0.0040 (11)	−0.0038 (11)	−0.0058 (13)
C7	0.0307 (12)	0.0381 (13)	0.0725 (18)	0.0016 (10)	0.0041 (11)	−0.0075 (13)
C8	0.0298 (11)	0.0317 (12)	0.0407 (13)	0.0015 (9)	0.0011 (10)	0.0009 (11)
C9	0.0313 (11)	0.0316 (12)	0.0401 (13)	−0.0004 (9)	0.0021 (9)	−0.0031 (11)
C10	0.0351 (12)	0.0303 (12)	0.0449 (13)	−0.0025 (10)	0.0030 (9)	−0.0032 (11)
C11	0.0336 (12)	0.0412 (13)	0.0804 (19)	−0.0008 (10)	0.0076 (13)	−0.0216 (15)
C12	0.0325 (13)	0.0431 (14)	0.0714 (18)	−0.0014 (11)	−0.0006 (12)	−0.0172 (14)
C13	0.0355 (11)	0.0323 (12)	0.0348 (12)	−0.0028 (10)	0.0041 (10)	−0.0037 (10)
C14	0.0348 (13)	0.0325 (12)	0.0390 (13)	0.0012 (9)	0.0060 (10)	0.0002 (11)
C15	0.0391 (14)	0.0436 (15)	0.083 (2)	0.0051 (11)	0.0043 (13)	−0.0157 (14)
C16	0.0546 (16)	0.0423 (14)	0.0667 (17)	0.0055 (12)	0.0074 (14)	−0.0140 (14)
C17	0.0456 (14)	0.0302 (12)	0.0426 (14)	−0.0028 (11)	0.0017 (11)	−0.0030 (11)
C18	0.0751 (17)	0.0528 (15)	0.0436 (14)	−0.0181 (14)	0.0089 (14)	0.0012 (12)
C19	0.0582 (15)	0.0452 (16)	0.0506 (15)	−0.0037 (12)	0.0074 (11)	−0.0088 (13)
C20	0.0523 (16)	0.0449 (16)	0.0460 (15)	−0.0017 (13)	0.0035 (12)	−0.0119 (13)
C21	0.073 (2)	0.0543 (17)	0.095 (2)	−0.0212 (15)	−0.0063 (17)	−0.0127 (16)

### Geometric parameters (Å, °)

**Table:**

Cl1—C4	1.747 (2)	C11—C12	1.536 (3)
O1—C3	1.212 (3)	C11—H11A	0.9700
O2—C20	1.338 (3)	C11—H11B	0.9700
O2—C17	1.454 (3)	C12—C13	1.521 (3)
O3—C20	1.196 (3)	C12—H12A	0.9700
C1—C2	1.533 (3)	C12—H12B	0.9700
C1—C10	1.532 (3)	C13—C18	1.525 (3)
C1—H1A	0.9700	C13—C17	1.533 (3)
C1—H1B	0.9700	C13—C14	1.534 (3)
C2—C3	1.485 (4)	C14—C15	1.528 (3)
C2—H2A	0.9700	C14—H14	0.99 (2)
C2—H2B	0.9700	C15—C16	1.534 (3)
C3—C4	1.473 (3)	C15—H15A	0.9700
C4—C5	1.337 (3)	C15—H15B	0.9700
C5—C6	1.502 (3)	C16—C17	1.531 (3)
C5—C10	1.526 (3)	C16—H16A	0.9700
C6—C7	1.522 (3)	C16—H16B	0.9700
C6—H6A	0.9700	C17—H17	1.03 (2)
C6—H6B	0.9700	C18—H18A	0.9600
C7—C8	1.521 (3)	C18—H18B	0.9600
C7—H7A	0.9700	C18—H18C	0.9600
C7—H7B	0.9700	C19—H19A	0.9600
C8—C14	1.519 (3)	C19—H19B	0.9600
C8—C9	1.537 (3)	C19—H19C	0.9600
C8—H8	0.97 (2)	C20—C21	1.496 (3)
C9—C11	1.535 (3)	C21—H21A	0.9600
C9—C10	1.572 (3)	C21—H21B	0.9600
C9—H9	1.03 (2)	C21—H21C	0.9600
C10—C19	1.535 (3)		
			
C20—O2—C17	118.21 (18)	C13—C12—C11	111.29 (19)
C2—C1—C10	112.97 (19)	C13—C12—H12A	109.4
C2—C1—H1A	109.0	C11—C12—H12A	109.4
C10—C1—H1A	109.0	C13—C12—H12B	109.4
C2—C1—H1B	109.0	C11—C12—H12B	109.4
C10—C1—H1B	109.0	H12A—C12—H12B	108.0
H1A—C1—H1B	107.8	C12—C13—C18	111.31 (18)
C3—C2—C1	111.1 (2)	C12—C13—C17	116.67 (18)
C3—C2—H2A	109.4	C18—C13—C17	108.70 (18)
C1—C2—H2A	109.4	C12—C13—C14	107.93 (16)
C3—C2—H2B	109.4	C18—C13—C14	112.67 (19)
C1—C2—H2B	109.4	C17—C13—C14	99.06 (16)
H2A—C2—H2B	108.0	C8—C14—C15	119.71 (19)
O1—C3—C4	122.3 (2)	C8—C14—C13	114.07 (16)
O1—C3—C2	122.6 (2)	C15—C14—C13	103.92 (17)
C4—C3—C2	115.0 (2)	C8—C14—H14	105.0 (12)
C5—C4—C3	124.8 (2)	C15—C14—H14	106.5 (12)
C5—C4—Cl1	121.81 (18)	C13—C14—H14	106.8 (12)
C3—C4—Cl1	113.38 (16)	C14—C15—C16	104.47 (19)
C4—C5—C6	122.3 (2)	C14—C15—H15A	110.9
C4—C5—C10	121.8 (2)	C16—C15—H15A	110.9
C6—C5—C10	115.94 (18)	C14—C15—H15B	110.9
C5—C6—C7	111.39 (18)	C16—C15—H15B	110.9
C5—C6—H6A	109.4	H15A—C15—H15B	108.9
C7—C6—H6A	109.4	C17—C16—C15	105.55 (18)
C5—C6—H6B	109.4	C17—C16—H16A	110.6
C7—C6—H6B	109.4	C15—C16—H16A	110.6
H6A—C6—H6B	108.0	C17—C16—H16B	110.6
C8—C7—C6	111.90 (19)	C15—C16—H16B	110.6
C8—C7—H7A	109.2	H16A—C16—H16B	108.8
C6—C7—H7A	109.2	O2—C17—C16	107.73 (18)
C8—C7—H7B	109.2	O2—C17—C13	114.86 (17)
C6—C7—H7B	109.2	C16—C17—C13	105.30 (19)
H7A—C7—H7B	107.9	O2—C17—H17	106.4 (12)
C14—C8—C7	111.91 (18)	C16—C17—H17	114.5 (12)
C14—C8—C9	108.23 (17)	C13—C17—H17	108.3 (11)
C7—C8—C9	110.17 (17)	C13—C18—H18A	109.5
C14—C8—H8	108.5 (12)	C13—C18—H18B	109.5
C7—C8—H8	109.9 (12)	H18A—C18—H18B	109.5
C9—C8—H8	108.1 (12)	C13—C18—H18C	109.5
C11—C9—C8	110.94 (17)	H18A—C18—H18C	109.5
C11—C9—C10	112.56 (17)	H18B—C18—H18C	109.5
C8—C9—C10	114.42 (17)	C10—C19—H19A	109.5
C11—C9—H9	105.5 (11)	C10—C19—H19B	109.5
C8—C9—H9	105.2 (11)	H19A—C19—H19B	109.5
C10—C9—H9	107.5 (11)	C10—C19—H19C	109.5
C5—C10—C1	110.32 (17)	H19A—C19—H19C	109.5
C5—C10—C19	109.15 (17)	H19B—C19—H19C	109.5
C1—C10—C19	110.36 (18)	O3—C20—O2	124.3 (2)
C5—C10—C9	107.58 (16)	O3—C20—C21	125.0 (2)
C1—C10—C9	107.54 (16)	O2—C20—C21	110.7 (2)
C19—C10—C9	111.85 (18)	C20—C21—H21A	109.5
C9—C11—C12	113.41 (19)	C20—C21—H21B	109.5
C9—C11—H11A	108.9	H21A—C21—H21B	109.5
C12—C11—H11A	108.9	C20—C21—H21C	109.5
C9—C11—H11B	108.9	H21A—C21—H21C	109.5
C12—C11—H11B	108.9	H21B—C21—H21C	109.5
H11A—C11—H11B	107.7		
			
C10—C1—C2—C3	−57.7 (3)	C11—C9—C10—C19	−59.0 (2)
C1—C2—C3—O1	−146.8 (2)	C8—C9—C10—C19	68.8 (2)
C1—C2—C3—C4	35.8 (3)	C8—C9—C11—C12	53.4 (3)
O1—C3—C4—C5	178.6 (2)	C10—C9—C11—C12	−176.93 (19)
C2—C3—C4—C5	−4.0 (3)	C9—C11—C12—C13	−54.1 (3)
O1—C3—C4—Cl1	−3.1 (3)	C11—C12—C13—C18	−69.5 (2)
C2—C3—C4—Cl1	174.23 (17)	C11—C12—C13—C17	164.98 (19)
C3—C4—C5—C6	170.6 (2)	C11—C12—C13—C14	54.6 (2)
Cl1—C4—C5—C6	−7.5 (3)	C7—C8—C14—C15	−54.6 (3)
C3—C4—C5—C10	−8.1 (4)	C9—C8—C14—C15	−176.23 (19)
Cl1—C4—C5—C10	173.84 (16)	C7—C8—C14—C13	−178.57 (18)
C4—C5—C6—C7	126.6 (2)	C9—C8—C14—C13	59.8 (2)
C10—C5—C6—C7	−54.7 (3)	C12—C13—C14—C8	−60.2 (2)
C5—C6—C7—C8	54.8 (3)	C18—C13—C14—C8	63.1 (2)
C6—C7—C8—C14	−175.36 (19)	C17—C13—C14—C8	177.88 (18)
C6—C7—C8—C9	−54.9 (3)	C12—C13—C14—C15	167.8 (2)
C14—C8—C9—C11	−54.1 (2)	C18—C13—C14—C15	−68.9 (2)
C7—C8—C9—C11	−176.8 (2)	C17—C13—C14—C15	45.8 (2)
C14—C8—C9—C10	177.19 (17)	C8—C14—C15—C16	−162.1 (2)
C7—C8—C9—C10	54.5 (3)	C13—C14—C15—C16	−33.5 (2)
C4—C5—C10—C1	−13.1 (3)	C14—C15—C16—C17	7.2 (3)
C6—C5—C10—C1	168.1 (2)	C20—O2—C17—C16	154.1 (2)
C4—C5—C10—C19	108.3 (2)	C20—O2—C17—C13	−88.9 (2)
C6—C5—C10—C19	−70.4 (2)	C15—C16—C17—O2	144.7 (2)
C4—C5—C10—C9	−130.1 (2)	C15—C16—C17—C13	21.7 (3)
C6—C5—C10—C9	51.1 (2)	C12—C13—C17—O2	85.0 (2)
C2—C1—C10—C5	45.1 (3)	C18—C13—C17—O2	−41.8 (2)
C2—C1—C10—C19	−75.5 (2)	C14—C13—C17—O2	−159.60 (18)
C2—C1—C10—C9	162.20 (19)	C12—C13—C17—C16	−156.7 (2)
C11—C9—C10—C5	−178.85 (19)	C18—C13—C17—C16	76.5 (2)
C8—C9—C10—C5	−51.0 (2)	C14—C13—C17—C16	−41.3 (2)
C11—C9—C10—C1	62.3 (2)	C17—O2—C20—O3	−0.1 (3)
C8—C9—C10—C1	−169.83 (18)	C17—O2—C20—C21	−179.48 (19)

### Hydrogen-bond geometry (Å, °)

**Table:**

*D*—H···*A*	*D*—H	H···*A*	*D*···*A*	*D*—H···*A*
C6—H6B···O1^i^	0.97	2.62	3.565 (3)	166.

Symmetry codes: (i) −*x*, *y*+1/2, −*z*+3/2.
